# Construction of a C-reactive protein-albumin-lymphocyte index-based prediction model for all-cause mortality in patients on maintenance hemodialysis

**DOI:** 10.1080/0886022X.2024.2444396

**Published:** 2025-01-14

**Authors:** Junmin Huang, Junfeng Hao, Huasheng Luo, Lu Chen, Hongying Luo, Huafeng Liu, Yongzhi Xu, Peng Wang

**Affiliations:** Department of Nephrology, National Clinical Key Specialty Construction Program (2023), Institute of Nephrology, Guangdong Provincial Key Laboratory of Autophagy and Major Chronic Non-communicable Diseases, Key Laboratory of Prevention and Management of Chronic Kidney Disease of Zhanjiang City, Affiliated Hospital of Guangdong Medical University, Zhanjiang, China

**Keywords:** C-reactive protein-Albumin-Lymphocyte Index, hemodialysis, mortality, prediction model

## Abstract

**Objective:**

The mortality rate of patients undergoing maintenance hemodialysis (MHD) remains high. The C-reactive protein-albumin-lymphocyte (CALLY) index is a novel biomarker that reflects inflammation, nutritional and immune status, all merged into one single derived parameter. No study has yet linked the CALLY index to survival in hemodialysis. This study aims to explore the correlation between the CALLY index and mortality in MHD patients, and develop and validate a nomogram to estimate the likelihood of death in this population.

**Methods:**

This retrospective cohort study collected data from 436 patients and they were divided into survival group (*n* = 335) and non-survival group (*n* = 101). Multivariate logistic regression analysis was used to screen factors associated with death, and nomograms were developed to estimate the risk of death in MHD patients. The discrimination and calibration of nomograms were validated using the area under the receiver operating characteristic (ROC) curve (AUC) and calibration curve. In the study, stratification analysis and covariate adjustment were conducted to explore the correlation between the CALLY index and the mortality of MHD patients.

**Results:**

In the final model, logistic regression showed that the CALLY index, creatinine, triglycerides, dialysis duration, absolute neutrophil count, blood urea nitrogen, sodium and ferritin were variables associated with mortality in MHD patients. A nomogram was developed to assess the risk of death in MHD patients. The AUC of the model was 0.821 (95% CI: 0.778–0.861). The results of stratified analysis and calibration model showed that the CALLY index was a protective factor for maintaining the mortality of MHD patients.

**Conclusions:**

The CALLY index is closely related to the mortality of MHD patients. A nomogram constructed based on CALLY index can effectively evaluate the mortality risk of MHD patients.

## Introduction

Chronic kidney disease (CKD) has emerged as an escalating global health concern. The 2017 global epidemiological survey reported an estimated 697.5 million individuals afflicted with CKD, representing a prevalence of 9.1% [1]. Maintenance hemodialysis (MHD) is the predominant therapeutic intervention for renal replacement therapy. In the updated review, HD prevalence continues to escalate annually, potentially attributed to earlier diagnosis and increased life expectancy [2]. However, the mortality rate among HD patients persists at a concerning high, fluctuating between 10% and 20% per annum. Meanwhile, HD patients have an uncontrolled high early mortality rate. Thus, most studies focus on the first year of dialysis [3]. At present, the risk factors that cause the mortality of hemodialysis patients to be higher than that of ordinary people have not been fully described. From the differences in age and complications, from planned dialysis to emergency dialysis, from biochemical indicators to dialysis adequacy, it is possible to affect the mortality rate of HD patients through different pathways. Current research attempts to find trajectories from different perspectives and identify high-risk populations. Furthermore, CVD has been identified as the predominant cause of mortality among individuals undergoing hemodialysis [4].

Microinflammatory state is a characteristic change in CKD and ESKD, usually characterized by persistent, low-grade elevation of noninfectious, circulating inflammatory markers. Persistent inflammatory signals can alter T cell phenotype and function, with increased production of pro-inflammatory cytokines and high circulating levels of TFH2 and plasmablasts [5]. In addition, as renal function deteriorates, the number of intermediate (CD14++ and CD16+) monocyte subpopulations increases [6]. The changes in immune function in HD patients are closely related to atherosclerosis or new tumors. CKD and ESRD patients commonly experience elevated catabolism of proteins and protein malnutrition, with potential causes including but not limited to metabolic acidosis, systemic inflammatory response, and accumulated uremic toxins [7]. Research shows that there is a negative correlation between the nutritional risk index and the risk of all-cause and cardiovascular death [8]. Based on the above observations, the malnutrition-inflammation-atherosclerosis syndrome has received attention in HD patients [9]. In addition, comparing the serum albumin trajectories of surviving patients undergoing HD, the albumin levels of deceased patients showed a downward trend and expanded with the progression of time [10]. The results underscore the importance of monitoring nutritional status in HD patients. The inflammatory composite index has shown considerable promise in predicting mortality. A study involving 787 patients revealed that a higher C-reactive protein/albumin ratio was significantly associated with an increased risk of death in HD patients within the first 6 months [11]. High platelet-to-lymphocyte ratio levels can independently predict all-cause mortality and cardiovascular mortality in MHD patients [12]. In the above context, inflammation, nutrition, and immunity, collectively referred to as modifiable risk factors, have attracted considerable attention in clinical decision-making for patients undergoing MHD.

The C-reactive protein-albumin-lymphocyte (CALLY) index, a novel biomarker reflecting inflammatory, nutritional, and immune status, is derived from serum levels of C-reactive protein, albumin, and lymphocyte count. It has been established as an independent risk factor in the diagnosis and prognosis of various malignancies, including colorectal and breast cancer, and is even superior to traditional factors [13–16]. Nonetheless, the correlation between the CALLY index and death in patients undergoing MHD has yet to be elucidated. This study hypothesizes that a comprehensive index reflecting inflammation, nutrition, and immune function may enhance the predictive accuracy of death in MHD patients. The present retrospective cohort study aims to investigate the association between the CALLY index and all-cause mortality, with the development and validation of a clinical prediction model utilizing the CALLY index to assess all-cause mortality in MHD patients, thereby facilitating the early identification of risk factors and enabling timely clinical intervention.

## Patients and methods

### Patient selection criteria and study design

This retrospective cohort study was conducted at the Affiliated Hospital of Guangdong Medical University, enrolling hemodialysis patients from 1 January 2018 to 31 December 2023.

As in previous studies, eligibility for the study was determined by the inclusion of adult hemodialysis patients aged 18 years or older who had undergone hemodialysis treatment for a minimum of 3 months [17]. Exclusion criteria were meticulously defined to ensure the study’s integrity: patients lacking complete baselines for the index of interest, receiving a kidney transplant, patients referred to the remaining centers for dialysis, those who had received peritoneal dialysis in conjunction with hemodialysis, patients with concurrent malignant tumors and patients suffering from acute infection were not included in the analysis. The hemodialysis protocol for the enrolled subjects entailed sessions scheduled two or three times weekly, with arteriovenous fistulas as vascular access.

**Figure 1.**
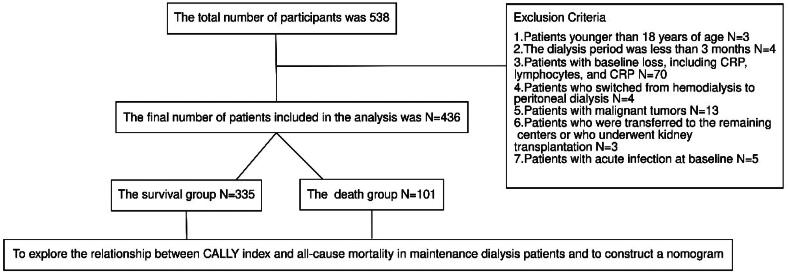
Flowchart showing the number of HD patients included in the analysis.

The endpoint events were all-cause death. For surviving patients, follow-up was until 31 December 2023 (Figure 1).

### Data collection and laboratory assessment

Demographic and clinical data were meticulously extracted from the electronic medical records, encompassing various parameters, including age, sex, comorbidities, dialysis period and pertinent laboratory indicators. 24-h urine-output was collected before regular hemodialysis. It is important to note that collection of laboratory data were taken for the first time after 3 months of stable hemodialysis treatment, with exclusions made for instances of acute infection. Except for the blood creatinine and urea nitrogen after dialysis, all blood samples were collected from fasting venous blood before dialysis. A comprehensive panel of biochemical variables was assessed, including creatinine, uric acid, hemoglobin, lymphocyte, neutrophils, blood platelets, calcium, sodium, potassium, phosphate, cholesterol, triglycerides, CO_2_, β2-microglobulin, intact parathyroid hormone (iPTH), single-pool *Kt*/*V* (spKT/V), ferritin, C-reactive protein, and albumin.

The interesting indicator in the article - CALLY index = (albumin × lymphocytes)/(C-reactive protein × 10). The spKT/V after dialysis was calculated using the following equation: SpKt/V = −ln [post-dialysis nitrogen/predialysis nitrogen −0.008 × treatment time] + [4–3.5 × post-dialysis nitrogen/predialysis nitrogen] × (predialysis weight - post-dialysis weight)/post-dialysis weight.

CVD refers to common diseases of the cardiovascular system that are cumulative in nature, excluding hypertension, including coronary heart disease, symptomatic arrhythmia, heart valve disease, heart failure, and cerebrovascular accidents.

### Statistical analyses

Statistical analyses were conducted rigorously, employing mean ± standard deviation (*SD*) for continuous variables and percentages for categorical variables. Univariate analyses were utilized to discern differences between groups, with the Student’s *t*-test applied to normally distributed data and the Mann-Whitney *U* test for non-normally distributed data. Categorical data were compared using the chi-square (*χ*^2^) test.

To select a model with excellent predictive performance based on the Akaike Information Criterion (AIC) principle and recognized clinical experience, we used stepwise backward logistic regression analysis to identify independent predictors of all-cause mortality. Perform stratified analysis on each layer to confirm the stability of the association between the CALLY index and all-cause mortality rate. An adjusted univariate logistic regression model was used to evaluate the association between the CALLY index and cardiovascular mortality. Statistical significance was defined as *p* < 0.05. All statistical analyses were performed using R version 4.2.3 and Python version 3.11.4.

### Model construction

The predictive model was constructed employing logistic regression analysis, with the binary outcome of interest being all-cause mortality after the initiation of hemodialysis. A backward stepwise logistic analysis based on the AIC principle was conducted to refine the model, and ultimately identifying the most significant predictors through multifactorial logistics.

Eventually, a nomogram predicated on the CALLY index was developed, encapsulating the significant predictors identified through multivariate analysis. This visual tool was designed to facilitate the estimation of all-cause mortality in hemodialysis patients, enhancing clinical decision-making.

### Model evaluation and internal validation

The discriminative power of the model was quantified by the area under the curve (AUC) of the receiver operating characteristic (ROC). Calibration was assessed using a calibration curve along with the Hosmer–Lemeshow test to evaluate the goodness-offit of the model.

To highlight the exceptional performance of the CALLY-based model (Model 1), we conducted a comparative analysis of its discriminative power and clinical utility against those of the albumin-based (Model 2), lymphocyte-based (Model 3), and CRP-based (Model 4) models.

The nomogram model was internally tested by bootstrap repeated sampling 1000 times, yielding an unbiased model performance estimate. Decision curve analysis (DCA) was employed to evaluate the clinical utility of the predictive models, illuminating whether the model’s application in clinical practice would confer a net benefit over existing strategies.

## Results

### Characteristics of patients undergoing hemodialysis with death

The study encompassed a total of 436 patients undergoing MHD, of which 23.16% (*n* = 101) encountered death of hemodialysis treatment initiation. The leading cause of death is CVD (*n* = 53, 52.47%). The cohort’s mean age was 59 (50, 68) years, with 61.7% (*n* = 269) being male. A stratified analysis at enrollment, based on the presence or absence of death, revealed significant demographic and clinical disparities. It is noteworthy that the patients in the death group were older (*p* < 0.001), had a shorter dialysis period (*p* = 0.014), and were more likely to have diabetes (*p* = 0.017) and CVD (*p* = 0.003). In addition, they had higher neutrophil count (*p* = 0.001), lower hemoglobin (*p* = 0.007), lower albumin (*p* < 0.001), higher C-reactive protein (*p* < 0.001), lower serum creatinine levels (*p* < 0.001), lower blood urea nitrogen (*p* = 0.027), lower sodium (*p* = 0.026), lower triglyceride levels (*p* = 0.002) and higher ferritin (*p* = 0.029). The baseline CALLY index of the deceased patients was significantly lower than that of the survivors (*p* < 0.001) (Table 1).

**Table 1.** Characteristics of patients undergoing hemodialysis with death.

Characteristic	Overall, *N* = 436^a^	Survival, *N* = 335^a^	Death, *N* = 101^a^	*p*-Value^b^
Sex				0.274
Male	269 (61.7%)	202 (60.3%)	67 (66.3%)	
Women	167 (38.3%)	133 (39.7%)	34 (33.7%)	
Age, years	59 (50, 68)	59 (48, 66)	65 (56, 74)	<0.001
Diabetes mellitus				0.017
No	293 (67.2%)	235 (70.1%)	58 (57.4%)	
Yes	143 (32.8%)	100 (29.9%)	43 (42.6%)	
Hypertension				0.868
No	23 (5.3%)	18 (5.4%)	5 (5.0%)	
Yes	413 (94.7%)	317 (94.6%)	96 (95.0%)	
Cardiovascular diseases				0.003
No	341 (78.2%)	273 (81.5%)	68 (67.3%)	
Yes	95 (21.8%)	62 (18.5%)	33 (32.7%)	
Dialysis duration, years	3.500 [1.417,4.833]	3.583 (1.667,4.917)	3.167 (0.833,4.583)	0.014
24-hour urine-output, mL	300.00 (0.00, 610.63)	320.00 (0.00, 662.50)	250.00 (0.00, 525.00)	0.244
Neutrophil count [absolute], 10^9^/L	4.31 (3.47, 5.27)	4.18 (3.44, 5.11)	4.75 (3.78, 5.76)	0.001
Platelet count, 10^9^/L	207 (161, 248)	208 (160, 254)	202 (165, 234)	0.325
Hemoglobin, g/L	112 (101, 119)	113 (102, 120)	107 (100, 119)	0.007
Lymphocyte count, 10^9^/L	1.12 (0.86, 1.42)	1.14 (0.86, 1.45)	1.05 (0.87, 1.34)	0.147
Albumin, g/L	40.10 (37.70, 42.20)	40.80 (38.70, 42.65)	38.30(36.30, 39.80)	<0.001
C-reactive protein, mg/L	5.00 (3.35, 7.77)	4.45 (3.03, 7.03)	7.02 (5.12, 10.20)	<0.001
Creatinine, μmol/L	912 (751, 1,063)	929 (795, 1,083)	836 (683, 945)	<0.001
Blood Urea Nitrogen, mmol/L	29 (24, 32)	29 (24, 33)	27 (23, 30)	0.027
Uric acid, mmol/L	462 (404, 529)	461 (406, 523)	471 (398, 547)	0.68
Sodium, mmol/L	138.30 (136.70, 139.78)	138.50 (137.00, 139.80)	137.90 (136.00, 139.30)	0.026
Potassium, mmol/L	4.91 (4.46, 5.32)	4.92 (4.47, 5.32)	4.89 (4.45, 5.29)	0.422
Calcium, mmol/L	2.25 (2.15, 2.39)	2.27 (2.14, 2.41)	2.23 (2.16, 2.35)	0.204
Phosphorus, mmol/L	2.10 (1.79, 2.46)	2.09 (1.80, 2.48)	2.12 (1.75, 2.35)	0.369
Parathyroid hormone, pg/ml	422 (254, 711)	426 (259, 731)	403 (241, 675)	0.393
Cholesterol, mmol/L	3.97 (3.32, 4.69)	3.98 (3.32, 4.67)	3.90 (3.35, 4.71)	0.778
Triglyceride, mmol/L	1.34 (0.97, 2.01)	1.39 (1.02, 2.12)	1.15 (0.92, 1.58)	0.002
Ferritin, μg/L	181 (90, 307)	175 (85, 288)	214 (115, 332)	0.029
Blood sugar, mmol/L	7.14 (6.11, 8.89)	7.21 (6.24, 8.76)	6.89 (5.87, 9.46)	0.442
β2 Microglobulin, mg/L	16.74 (14.58, 18.88)	16.78 (14.69, 18.84)	16.23 (14.23, 18.56)	0.993
CO_2_, mmHg	19.00 (17.00, 20.80)	19.0 (17.00, 20.90)	18.9 (17.00, 20.50)	0.647
SpKT/V	1.43 (1.25, 1.64)	1.43 (1.25, 1.64)	1.42 (1.22, 1.67)	0.657
CALLY index	0.89 (0.55, 1.37)	1.02 (0.65, 1.62)	0.57 (0.43, 0.83)	<0.001

### Association of all-cause mortality with CALLY index and other variables in patients undergoing hemodialysis

To avoid collinearity, we did not choose to include albumin, lymphocyte count, and C-reactive protein for analysis. The principle of this experiment is to pick the model combination with the smallest AIC. The final model (Model 1) obtained by stepwise regression included creatinine, triglycerides, dialysis duration, absolute neutrophil number, blood urea nitrogen, sodium, ferritin, and CALLY index, although some of the *p* values were not less than 0.05 (Table 2).

**Table 2.** Univariate and multivariate logistic regression analysis of the result of death and clinical candidate predictors.

	Univariate analysis		Multivariate analysis	
Variables	OR (95% CI)	*p*	OR (95% CI)	*p*
Age	1.034 (1.017–1.052)	<0.001	–	–
Cardiovascular disease	2.137 (1.297–3.519)	0.003	–	–
Diabetes mellitus	1.742 (1.101–2.756)	0.018	–	–
Dialysis duration	0.872 (0.773–0.984)	0.026	0.886 (0.765–1.025)	0.104
Neutrophil count	1.302 (1.108–1.532)	0.001	1.235 (1.027–1.491)	0.026
Creatinine	0.998 (0.997–0.999)	<0.001	0.999 (0.997–1.000)	0.036
Triglycerides	0.706 (0.541–0.920)	0.01	0.659 (0.486–0.858)	0.004
CALLY index	0.149 (0.082–0.273)	<0.001	0.153 (0.077–0.283)	<0.001
Hemoglobin	0.979 (0.964–0.994)	0.007	–	–
Blood Urea Nitrogen	0.957 (0.923–0.992)	0.015	0.967 (0.921–1.005)	0.17
Sodium	0.898 (0.823–0.979)	0.015	0.936 (0.846–0.994)	0.193
Ferritin	1.001 (1.000–1.002)	0.02	1.001 (1.000–1.002)	0.079
Uric acid	1.000 (0.998–1.003)	0.758	–	–

**Figure 2.**
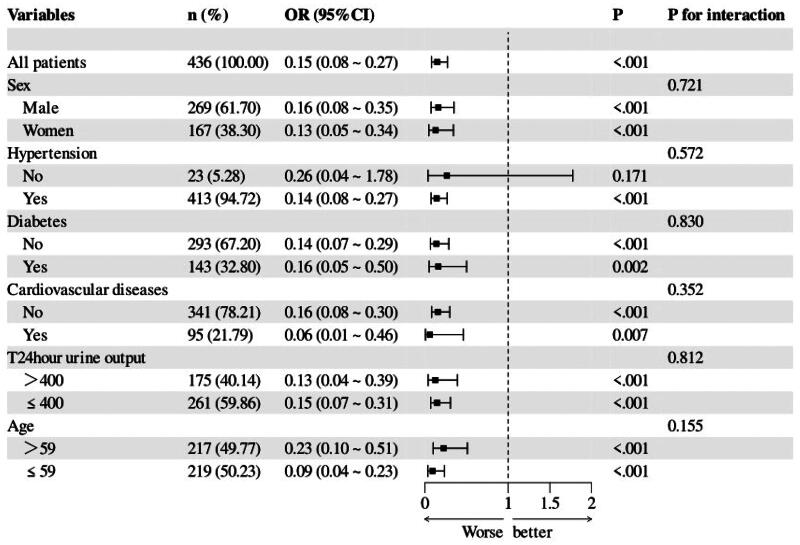
The association between the CALLY index and all-cause mortality in HD patients with different stratification levels.

We further explored the correlation between the CALLY index and all-cause mortality. Stratified analyses showed that the correlation between the CALLY index and all-cause mortality was stable in all stratified groups, except in those without hypertension (*p* = 0.171), with an interaction of *p* > 0.05 in all cases. (Figure 2). For the CALLY index only, we adjusted different covariates for model a (containing sex, age), model b (containing sex, age, hypertension, diabetes, CVD dialysis period, and 24-h urine-output), and model c (containing sex, age, hypertension, diabetes, CVD, dialysis period, 24-h urine-output and all the tests). In all adjusted models, the CALLY index was a protective factor for all-cause mortality (*p* < 0.001) (Table 3).

**Table 3.** The model was adjusted by adding covariates to further validate the robustness of the CALLY index in association with all-cause mortality in MHD patients.

Characteristic	Model a				Model b				Model c			
	*N*	OR^a^	95% CI^a^	*p*-Value	*N*	OR^a^	95% CI^a^	*p*-Value	*N*	OR^a^	95% CI^a^	*p*-Value
CALLY index (continuous)	436	0.167	0.090, 0.309	<0.001	436	0.152	0.080, 0.288	<0.001	436	0.179	0.089, 0.360	<0.001

^a^OR: Odds Ratio; CI: Confidence Interval.

Model a: adjusted for sex and age.

Model b: adjusted for sex, age, hypertension, diabetes, CVD, dialysis period, and 24-h urine-output.

Model c: adjusted for sex, age, hypertension, diabetes, CVD, dialysis period, 24-h urine-output, neutrophils, platelet, hemoglobin, creatinine, urea nitrogen, uric acid, sodium, potassium, calcium, phosphorus, cholesterol, triglyceride, blood sugar, CO_2,_ ferritin, β 2 microglobulin, parathyroid hormone and spKT/V.

For this purpose, we performed additional analyses, and the CALLY index was likewise negatively associated with cardiovascular death in patients on maintenance hemodialysis (OR = 0.202, *p* < 0.001). The same conclusion was reached after adjusting for all confounders (OR = 0.239, *p* = 0.002) (Table 4).

**Table 4.** The model was adjusted by adding covariates to further validate the robustness of the CALLY index in association with cardiovascular death in MHD patients.

Characteristic	Model *d*				Model e			
	*N*	OR^a^	95% CI^a^	*p*-Value	*N*	OR^a^	95% CI^a^	*p*-Value
CALLY index (continuous)	436	0.202	0.096, 0.422	<0.001	436	0.239	0.097 0.592	0.002

^a^OR: Odds Ratio; CI: Confidence Interval.

Model d: no adjusted.

Model e: adjusted for sex, age, hypertension, diabetes, CVD, dialysis period, 24-h urine-output, neutrophils, platelet, hemoglobin, creatinine, urea nitrogen, uric acid, sodium, potassium, calcium, phosphorus, cholesterol, triglyceride, blood sugar, CO_2,_ ferritin, β 2 microglobulin, parathyroid hormone and spKT/V.

### CALLY index-based nomogram development and validation

**Figure 3.**
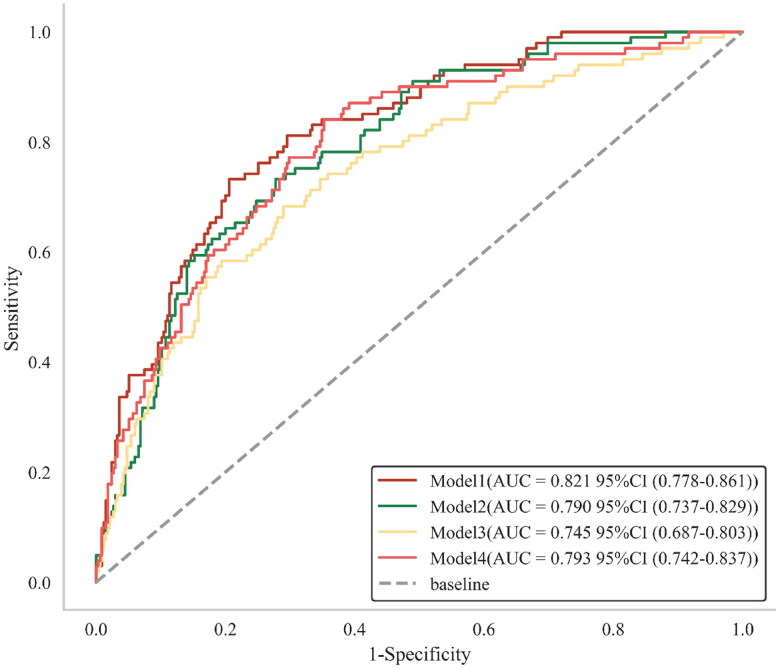
Comparison of four models’ discriminative abilities. Model 1: CALLY index + creatinine + triglycerides + dialysis duration + neutrophils + blood urea nitrogen + sodium + ferritin, Model 2: albumin + creatinine + triglycerides + dialysis duration + neutrophils + blood urea nitrogen + sodium + ferritin, Model 3: lymphcyte + creatinine + triglycerides + dialysis duration + neutrophils + blood urea nitrogen + sodium + ferritin, Model 4: CRP + creatinine + triglycerides + dialysis duration + neutrophils + blood urea nitrogen + sodium + ferritin.

**Figure 4.**
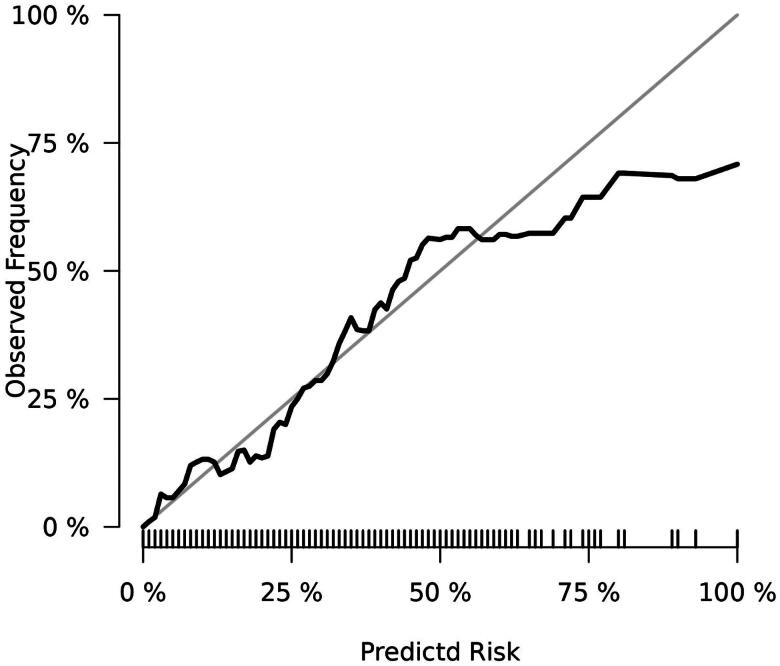
The calibration curve of the model1. Within the range of actual probability of death in hemodialysis patients, the prediction accuracy of the model was relatively overlapped.

The final prediction model (Model 1) contained 8 variables, including creatinine, triglycerides, dialysis period, neutrophils, urea nitrogen, sodium, ferritin, and CALLY index. Model 1, with an AUC of 0.821 (95% CI:0.778–0.861), outperforms Model 2 with an AUC of 0.790 (95% CI:0.737–0.829), as well as Model 3 with 0.745 (95% CI:0.687–0.803), and Model 4 with 0.793 (95% CI:0.742–0.837) (Figure 3). Among all the constructed prediction models, Model 1 showed the best specificity and accuracy (Table 5). Furthermore, the calibration curve suggests appropriate agreement between the model prediction and actual observations in the dataset, with a *p*-value of 0.411 for the Hosmer-Lemeshow test (Figure 4).

**Table 5.** Multi-angle comparison of the prediction performance of four models.

	*N*	AUC	Sensitivity	Specificity	Youden index	Diagnostic threshold	Accuracy
Model 1	436	0.821 (0.778–0.861)	0.733 (0.671–0.874)	0.794 (0.669–0.830)	0.527 (0.457–0.616)	0.306 (0.206–0.307)	0.764 (0.706–0.808)
Model 2	436	0.790 (0.737–0.829)	0.733 (0.580–0.951)	0.722 (0.499–0.863)	0.455 (0.384–0.564)	0.227 (0.139–0.328)	0.725 (0.603–0.813)
Model 3	436	0.745 (0.687–0.803)	0.683 (0.531–0.828)	0.710 (0.570–0.859)	0.394 (0.348–0.517)	0.246 (0.200–0.312)	0.722 (0.614–0.815)
Model 4	436	0.793 (0.742–0.837)	0.842 (0.748–0.933)	0.648 (0.569–0.771)	0.489 (0.431–0.607)	0.195 (0.154–0.237)	0.702 (0.646–0.791)

Among them, model 1 had the best specificity and accuracy, and its diagnostic threshold was 0.306.

**Figure 5.**
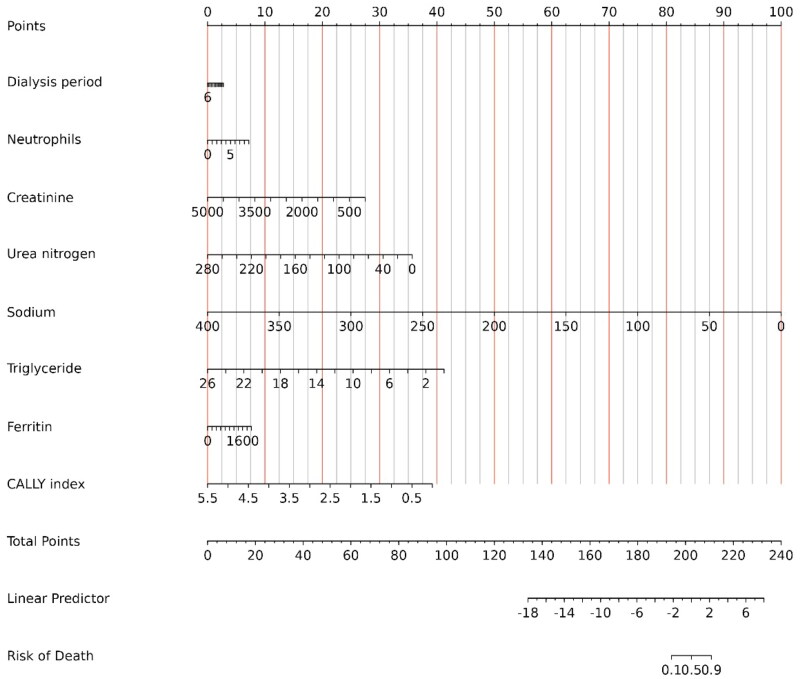
Discrimination of the CALLY index-based nomogram for All-Cause Mortality determining.

**Figure 6.**
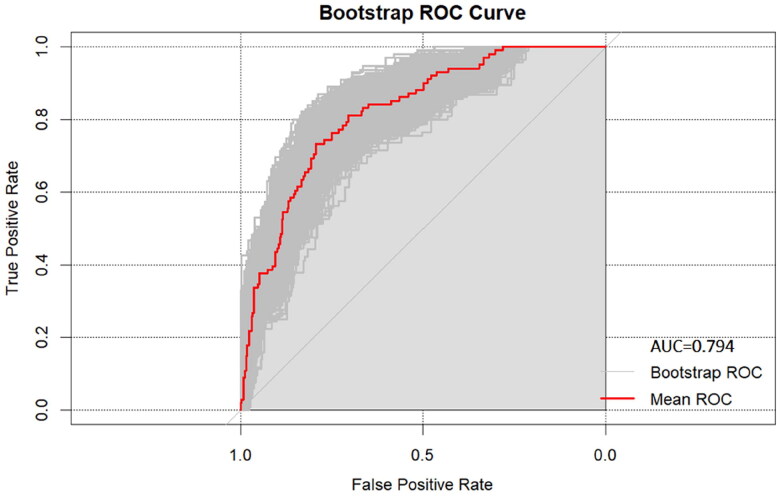
The Bootstrap results of Model 1, with repeated sampling *N* = 1000.

Combining the above 8 predictive factors, a nomogram based on the CALLY index was developed to evaluate the all-cause mortality rate of MHD patients (Figure 5). After 1000 bootstrap internal validations, the ROC curve showed a mean AUC of 0.794, indicating the model has repeatability in this dataset and appropriate discriminative power (Figure 6).

**Figure 7.**
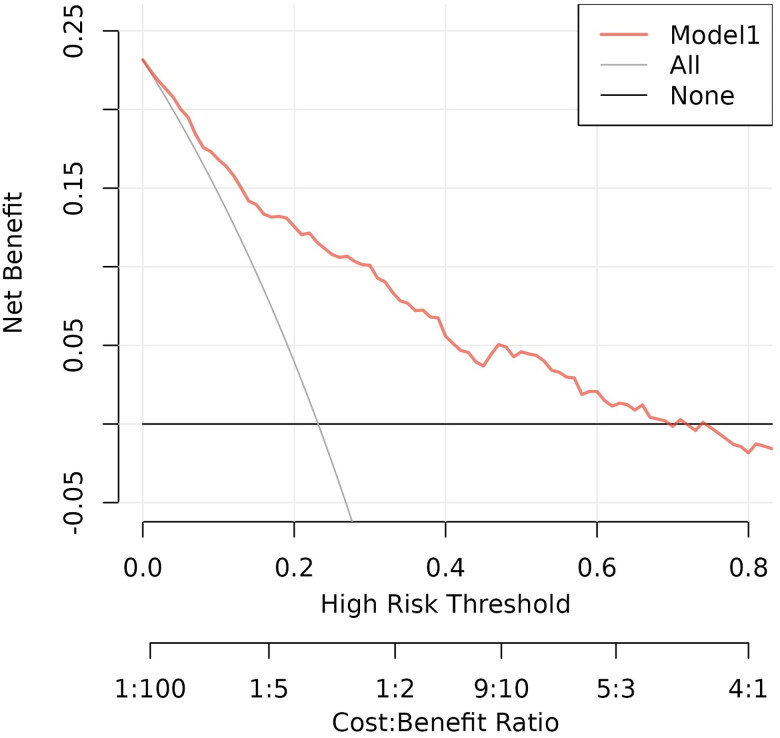
Decision curve for the Model 1. The all-cause mortality rate for MHD patients is 23.17%, and the model has practical clinical value in this range.

Our results showed an all-cause mortality rate of 23.17%, which is slightly higher than reported in the literature. Within the risk threshold 0–0.7, the decision curve is located above the None lines and All lines; therefore, the Model 1 can be considered clinically practical (Figure 7).

## Discussion

Despite the current development of hemodialysis technology, the hospitalization and mortality rates of MHD patients are still not ideal. Cardiovascular events are the leading cause of death in MHD patients. Early CKD patients may have atherosclerosis and calcification, and their clinical manifestations are often hidden but accompanied by acute attacks [18]. The above mentioned that in every country, the mortality rate in the early stages of hemodialysis is higher than in the middle period [1]. It is essential to identify risk factors and develop death prediction models to improve the survival of MHD patients. Therefore, further identifying effective biomarkers for predicting death in MHD patients is necessary.

The CALLY index comprises CRP, serum albumin, and lymphocytes, representing inflammation levels, nutritional status, and immune function. Our study found a correlation between the CALLY index and all-cause mortality in MHD patients through multivariate analysis for the first time. The CALLY index is a protective factor for death. Our study encompassed patients undergoing dialysis twice weekly and thrice weekly, among whom there were certain differences in health status and levels of residual renal function. In this respect, 24-h urine volume was employed to evaluate the level of residual renal function. 24-h urine volume and complications were taken as confounding factors in the model adjustment and stratified analyses to further guarantee the reliability of the results. Even in the models adjusting for confounding factors, the above correlation is still evident. The results of the stratified analysis showed a stable association between the CALLY index and mortality, except for those without high blood pressure. We further derived a negative association between CALLY and cardiovascular death, which, interestingly, has also not been mentioned in the previous literature.

CALLY as a predictor of all-cause and cardiovascular death is consistent with theory. CRP is not only a marker of CVD and death but also a participant in a pathogenic way, including but not limited to (a) the combination of CRP and degraded LDL can enhance complement activation and induce the expression of tissue factors [19]; (b) CRP participates in the formation of vascular calcification by promoting the phenotype transformation of vascular smooth muscle into osteoblasts [20]; (c) CRP induces and recruits monocytes in early atherosclerosis through specific CRP receptors [21]. Previous studies have shown that high sensitivity CRP levels at baseline independently predict all-cause mortality [22]. Moreover, Serum albumin is an independent risk factor for cardiovascular mortality in MHD patients, which may be explained by inflammation and nutritional pathways [23]. The albumin binding and detoxification efficiency after HD are significantly lower than those of healthy individuals [24]. MHD patients are in a long-term weakened state, and reasonable nutritional intervention will improve most of the weakened or pre-weakened states [25]. During the progression of CKD, lymphocytes undergo depletion and subgroup changes, including early and persistent adverse effects [26]. However, as a high-risk population for cardiovascular events associated with various risk factors, the mechanisms underlying changes in the number and function of immune cells in MHD patients have not been fully elucidated. Research shows that in hemodialysis patients, the activation of T cells tends to Th1 rather than Th2, and Th1 cells can promote atherosclerosis, which may be the leading cause of disease [27,28]. In our results, the CALLY index worked better as part of the predictors than either albumin, C-reactive protein, or lymphocytes added to the model. In conclusion, the CALLY index is an excellent composite indicator.

All-cause mortality rate of our study was 23%, which is not completely consistent with previous research reports and may be related to advanced age [29,30]. Through stepwise backward logistic regression analyses, we included common and established traditional risk factors in our final model. However, the above results do not represent the failure of other risk factors. Research has shown a U-shaped relationship between serum potassium and death or the onset of KRT in CKD grade 4–5 patients [31]. Moreover, patients in the death group were found to have lower creatinine levels in our center, and acted as a protective factor for death, although the association was weak. This is not surprising, however, as it has been shown that the link between creatinine and death is not simply linear but has a U-shaped distribution [32]. In line with previous studies, a significant disparity was observed in the years of dialysis between the two groups of patients, indicating that patients with a longer duration of dialysis should be monitored more closely [33]. Additionally, our study included ferritin as a predictor variable. A multicenter study has proposed that different regions have different optimal iron doses and anemia management, and emphasized the association between adjusting nutrition and inflammation. This further emphasizes the necessity of center-specific prediction models [34]. The exclusion of patients on peridialysis due to the high mortality rate during this period might have been insufficient to eliminate the associated systematic bias. To this end, we can only guarantee the stability of our experiments and avoid the remaining biases through more detailed inclusion and exclusion criteria.

As far as we know, no research CALLY index predicts the effectiveness of mortality. This predictive model in the present study is the first nomogram constructed based on the CALLY index to predict all-cause mortality in MHD patients. The AUC results indicate that the model has confident predictive performance and has the same results in internal validation. The DCA curve suggests that the model has clinical efficacy within the 15–22% range of mortality in MHD patients proposed in the literature. The model we constructed is easy to obtain and can serve as a preliminary screening method for all-cause mortality in MHD patients, but some limitations should still be considered. Firstly, the sample size included is relatively small. Secondly, the potential risk factors we included in the study were insufficient, excluding more complications associated with CKD, medication regimens, smoking, BMI, and more comprehensive biochemical indicators. The aforementioned factors might contribute to the model construction and mitigate the bias of the correlation. Thirdly, our research is based on a static prediction model constructed from baseline data without linking the dynamic changes in data with outcome events. Fourthly, there is a lack of another independent dataset for external validation of the model to verify whether our results have universal applicability. Furthermore, our model seems to be less suitable for the acute phase of the disease, especially in the acute infection stage, although intervening in indicators that do not show significant changes is also one of the design goals.

## Conclusion

We propose for the first time the correlation of the CALLY index with mortality in patients on MHD and provide a new perspective on the prognosis of other diseases. Our main goal is to intervene in biochemical indicators during the stable period of patients, which is an active process rather than a passive process that only begins when the condition progresses. In conclusion, based on the CALLY index, we developed a predictive model for all-cause mortality in MHD patients, which has good predictive power and clinical application value. We believe this model will help improve MHD patients’ quality of life and survival rate.
